# Base damage, local sequence context and *TP53* mutation hotspots: a molecular dynamics study of benzo[a]pyrene induced DNA distortion and mutability

**DOI:** 10.1093/nar/gkv910

**Published:** 2015-09-22

**Authors:** Georgina E. Menzies, Simon H. Reed, Andrea Brancale, Paul D. Lewis

**Affiliations:** 1 Institute of Life Science, Swansea University School of Medicine, Swansea University, SA2 8PP, UK; 2 Institute of Cancer & Genetics, School of Medicine, Cardiff University, CF14 4XN, UK; 3 School of Pharmacy and Pharmacology, Cardiff University, CF10 3NB, UK

## Abstract

The mutational pattern for the *TP53* tumour suppressor gene in lung tumours differs to other cancer types by having a higher frequency of G:C>T:A transversions. The aetiology of this differing mutation pattern is still unknown. Benzo[a]pyrene,diol epoxide (BPDE) is a potent cigarette smoke carcinogen that forms guanine adducts at *TP53* CpG mutation hotspot sites including codons 157, 158, 245, 248 and 273. We performed molecular modelling of BPDE-adducted *TP53* duplex sequences to determine the degree of local distortion caused by adducts which could influence the ability of nucleotide excision repair. We show that BPDE adducted codon 157 has greater structural distortion than other *TP53* G:C>T:A hotspot sites and that sequence context more distal to adjacent bases must influence local distortion. Using *TP53* trinucleotide mutation signatures for lung cancer in smokers and non-smokers we further show that codons 157 and 273 have the highest mutation probability in smokers. Combining this information with adduct structural data we predict that G:C>T:A mutations at codon 157 in lung tumours of smokers are predominantly caused by BPDE. Our results provide insight into how different DNA sequence contexts show variability in DNA distortion at mutagen adduct sites that could compromise DNA repair at well characterized cancer related mutation hotspots.

## INTRODUCTION

*TP53* is a tumour suppressor gene that encodes the transcription factor p53 with anti-proliferative functions in response to cellular stress (1). Located on chromosome 17p13, *TP53* is the most commonly mutated gene in human cancer where the somatic mutation frequency can be as high as 80% in some tumour types (2). For lung cancer, approximately 50% of tumours contain a *TP53* mutation. The mutational pattern in lung tumours differs from most other cancer types by having a higher frequency of G:C>T:A transversions (3–5). The aetiology of this differing mutation spectral pattern has been extensively debated (6). It is agreed however, that *TP53* mutations likely result due to the formation of adducts by polycyclic aromatic hydrocarbons (PAH), from cigarette smoke, as well as oxidatively induced lesions including hydroxyl radicals (OH˙) (7–9).

**Figure 1.**
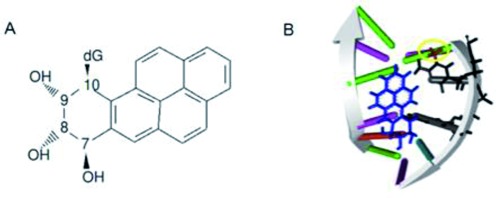
Chemical structure and mutagenic adduct formation of Benzo(a)pyrene diolepoxide. (**A**) Chemical structure of 7S,8R,dihydroxy-9R,10S-epoxy-7S,8R,9R,10S (+)-trans-anti-B[a]PDE; (**B**) adduct of BPDE (blue) bound to guanine in a CpG site in minor groove directed towards the 3′ end of the DNA sequence. Also shown is the methyl group (highlighted by a yellow circle) on the cytosine. The adducted guanine ‘G’ and 5′ adjacent methylated cytosine ‘C’ are indicated by arrows.

Benzo[a]pyrene, a PAH, is a potent carcinogen, produced by incomplete combustion and mutagen found in cigarette smoke. Benzo[a]pyrene is metabolized in cells to the carcinogenic derivative trans(+)anti-benzo[a]pyrene diol expoxide (BPDE). Benzo[a]pyrene is found in cigarette smoke but also present in the environment, e.g. in exhaust fumes. Smoking one cigarette is known to yield an intake of 20–40 ng of benzo[a]pyrene (10). A study by Goldman *et al*., concluded that smoking increased the concentration of benzo(a)pyrene, ∼2-fold in the lungs of patients analysed (11). There is empirical evidence for a strong correlation between adduct positions of BPDE (Figure 1) and *TP53* base substitution hotspots in smokers (12,13). BPDE binds to the N2 position of guanine and Kozack and Loechler *et al.*, report eight probable adduct conformations with five of these thought to be relevant to mutagenesis (14). One of these adducts, 7S,8R,dihydroxy-9R,10S-epoxy-7S,8R,9R,10S (+)-trans-anti-B[a]PDE, is suggested to be the most mutagenic form where BPDE sits in the minor groove directed towards the 5′ end of the DNA sequence (15).

Mutagen target specificity and compromised DNA repair are the major factors contributing to the likelihood of a mutation arising in a DNA sequence. Selection dictates whether the mutation will be observed and the strength of selection can influence the shape of the mutation spectrum for *TP53* in a given cancer. Sequence context influences both the rate of mutagen binding and the rate of lesion repair of adducts including those for BPDE by nucleotide excision repair (NER) (16–18). Binding affinity of BPDE with guanines at *TP53* mutation hotspot CpG sites has been confirmed at codons 157, 158, 245, 248, 273 and 282 (12). These, and other studies, also showed that BPDE targets as well as causes G:C>T:A substitutions at CpG sites such as in codons 170, 186, 213, 267 and 290 but these are either uncommon or silent mutation sites in cancer (12,19–21). Interestingly, mutation hotspots at codons 157 and 158 are common in lung tumours but not other tumour types. Given the wide BPDE target specificity across codons and accounting for selection then, if BPDE causes mutations in *TP53*, there is likely a combination of differential adduct levels and repair capacity across adduct sites.

The rate by which NER excises lesions in different sequence contexts is known to vary greatly (22–25). Empirical data have revealed slow repair of BPDE at *TP53* codons 157, 248, 273 and 282 but not others (19). This provides weight to the hypothesis that BPDE causes mutations at these sites in the lung cancer spectrum. The reason why slow repair occurs at these sites and not others is, however, unknown but a number of factors could be considered. Methylation at CpG sites impacts upon the local DNA flexibility and *TP53* CpG sites are methylated in the lung (20,26). Derreumaux *et al*. propose that the reduction of DNA flexibility by CpG methylation impacts on the DNA's repair rate when compared to the same sequence without methylation (27). As all *TP53* CpG sites are methylated in the lung, methylation alone cannot account for variable DNA repair rates at BPDE binding sites thus one must consider other sequence-context dependent structural parameters.

Many studies, both experimental and theoretical, show the importance of DNA distortions such as kinks, hydrogen-bonding and flipped nucleotides for recognition by NER, but the contribution leading to mutagenesis is unclear (28–31). Structural parameters such as the amount of sequence dependent twist or un-twist in a DNA helix also affect DNA repair rate due to differing protein binding ability (16–17,32). Thus, it is possible that repair capacity in *TP53* is variably compromised across BPDE adduct sites by differing changes in DNA structural parameters according to sequence context. To date however, there has been no attempt to determine the correlations between DNA sequence context, local structural distortions caused when adducts are present and the pattern of mutation at hotspot sites in *TP53*.

In this study, we used molecular modelling of known BPDE adducted *TP53* duplex sequences (six mutation hotspot sites and five non-hotspot sites) with Molecular Dynamics (MD) simulations to determine the degree of local distortion caused by adducts. We were motivated by the fact that no previous simulated structural distortion data have been generated for mutation's hotspots in the *TP53* gene. Our overall goal was to assess adducted-induced structural distortion in relation to sequence context at each site. This could help explain why certain mutation hotspots occur in lung cancer of smokers and provide hypotheses about how NER might be compromised at certain sequences. Adduct-induced distortion for each sequence was determined using a range of DNA helical parameters. The simulations provided data on the degree of intra- and inter-base pair rotational and translational movements as well as base pair axis parameters at these adduct sites after a fixed time. Using multivariate statistical analysis we were able to interpret the relative distortion caused by adducts between damaged sequences as well as to the same non-adducted sequences. The modelling revealed that BPDE adducted codon 157 has a more severe DNA structural distortion than other smoking related lung cancer *TP53* G:C>T:A hotspot sites including codons 158, 245, 248 and 273. As codons 157 and 273 have the same 5′ and 3′ neighbouring bases we conclude that sequence context more distal to adjacent bases must be responsible for the differing structural distortion at these two sites.

Using *TP53* mutation signatures for lung cancer in smokers and non-smokers, we then assessed the mutation frequency at each modelled site in relation to local sequence context involving 5′ and 3′ adjacent bases. The G:C>T:A mutation frequency data revealed that the CGT/ACG trinucleotide, as observed at codons 157 and 273, has the highest mutation probability in smokers. The data also showed that the probability is increased in CGG/CCG as well as CGT/ACG sites in smokers relative to non-smokers. With increased adduct-induced structural distortion at codon 157, we predict that G:C>T:A mutations observed in lung tumours of smokers are predominantly caused by BPDE. Our results provide insight into how different DNA sequence contexts show variability in DNA distortion at mutagen adduct sites that could compromise DNA repair at well characterized cancer related mutation hotspots.

## MATERIALS AND METHODS

### *TP53* DNA sequences

MD simulations were performed on eleven 11-mer *TP53* duplex DNA sequences encompassing either mutation hotspot or non-hotspot codons in lung cancer (Table 1). Each sequence has a methylated cytosine and a guanine at the fifth and sixth nucleotide positions, respectively. The guanine at position six in each sequence is the mutation site for any hotspot sequence. The guanines at position six in every sequence have also been shown to be strong or weak BPDE binding sites (12). Simulations were performed on both adducted and non-adducted sequences. A sequence was designated as containing a lung cancer mutation hotspot at position six guanine if the number of G:C>T:A substitutions recorded at this site in the IARC *TP53* database (33) was significantly increased relative to expected (34). Briefly, in the IARC *TP53* database there are 421 recorded G:C>T:A substitutions in exons 5–8 for lung tumours where the smoking status was known. In *TP53* exons 5–8 there are 353 guanines, i.e. possible mutable sites. If one assumes that mutations are equally likely in every guanine then the distribution of G:C>T:A substitutions across all guanines will follow a multinomial distribution with a probability that 1/353 mutations would occur at each site (*P*). A guanine is classified as a mutation hotspot if the number of mutations (*N*) at the site is in excess of that predicted by a binomial distribution with probability *P*. The probability of finding *N* G:C>T:A substitutions at a site was calculated using an exact binomial test and compared to *P* with a Bonferroni correction. Codons with significant mutation hotspots for lung cancer in smokers were 157, 158, 245, 248 and 273 (Table 1). Codons 213 and 290 each had two G:C>T:A substitutions recorded but were not significant hotspots. Codon 282 had a single G:C>T:A substitution and codons 170, 186, 267 have no mutations recorded and are thus not hotspots. The same method was applied to determine whether codons were significant mutation hotspots in other cancers.

**Table 1.** *TP53* DNA sequences used for molecular dynamics simulations (adducted guanine underlined). Sequences were determined to be hotspots in cancers based on their prevalence in the IARC *TP53* database, or the UMD *TP53* database (5,33)

CODON	SEQUENCE	HOTSPOT STATUS (across cancers)	No. observed G:C>T:A in lung cancer *	*P*-value **
157	CCCGCGTCCGC	Lung	27	1 x 10^−27^
158	CGTCCGCGCCA	Lung	35	4 x 10^−39^
245	TGGGCGGCATG	Many	9	4 x 10^−6^
248	GAACCGGAGGC	Many	24	2 x 10^−23^
273	GGTGCGTGTTT	Many	36	1 x 10^−40^
282	AGACCGGCGCA	Many	1 (NS)	0.362
170	ATGACGGAGGT	None	0	0.303
186	ATAGCGATTGT	None	0	0.303
213	TTTTCGACATA	Breast	2 (1 NS)	0.216
267	GGGACGGAACA	None	0	0.303
290	TCTCCGCAAGA	None	2 (1 NS)	0.216

*Total number of observed G:C>T:A substitutions observed in the IARC *TP53* database for codons 5–8 where smoking status was known.

**P-value denotes for each codon whether the number of G:C>T:A substitutions at the mutable guanine formed a significant mutation hotspot in lung cancer.

NS = Non-smoker.

### Starting structures and force field

The starting structures for each 11 base pair (bp) unmodified DNA duplex were built using the Discovery Studio v3.5(Accelrys Software, Inc.). A methylated group was then added to the fifth (cytosine) base also using the nucleic acid tools provided within Discovery studio v3.5. These non-adducted control sequences were all geometry optimized within the Molecular Operating Environment (MOE) (Chemical Computing Group), using the amber99 force field. No nuclear magnetic resonance (NMR) structures were available for all sequences required; however, a NMR structure for the 7S,8R,dihydroxy-9R,10S-epoxy-7S,8R,9R,10S (+)-trans-anti-B[a]PDE DNA adduct in a DNA sequence of the required length was available from the Protein Databank (PDB ID: 1AXO) (35). This PDB file contains coordinates for six structures obtained from a relaxation matrix refinement and the first structure was chosen. The sequence obtained from that structure was remodelled to the sequences of the *TP53* hotspots and non-hotspots using the modelling software, MOE and Discovery Studio v3.5 following previously published methods (36). Each nucleotide was changed as appropriate using Discovery Studio v3.5, and minimized using the parm99 force field built into MOE providing structures with BPDE adducts at the sixth nucleotide guanine.

The partial charges for the modified bases (BPDE adducted guanine and methylated cytosine) were calculated using MOE and the force field used to calculate these charges was Amber99 (37). Bond and angle parameters were assigned for the adducted nucleotides and added to the Amber99 force field found within GROMACS v4.5 (38). These parameters were assigned based upon chemically similar existing parameters, with the help of the Ambertools module (37).

### Molecular dynamics (MD) simulations

All MD simulations were carried out using the GROMACS 4.5 package (38) using the Amber99 force field. The DNA structures were boxed and solvated using the GROMCAS module. The DNA molecule was placed in the centre of a cubic box and solvated using single point charge water molecules, SPC216. The box surrounding the molecule was approximately 6 nm in length and filled with ∼7500 water molecules (with some variation between simulations). In order to neutralize the system, a total of 20 Na+ ions were added to the box in the place of 20 water molecules. The particle mesh ewald (PME) method was used to treat long-range electrostatic interactions and a 1.4 nm cut-off was applied to Lennard–Jones interactions.

The MD simulations were all carried out in the NPT ensemble, with periodic boundary conditions, at a temperature of 300°C, and a pressure of 1 atm. Each simulation was performed using a three-step process: steepest descent energy minimization with a tolerance of 1000 KJ^−1^ nm^−1^; a pre MD run (PR) with 25 000 steps at 0.002 per second per step making a total of 2500 ps; an MD stage run for a total of 10 ns. Root mean square deviation (RMSD) was monitored along with the total energy, pressure and volume of the simulation in order to monitor the stability of the simulations.

### Hydrogen bond quality index

We used the Hydrogen Bond Quality Index to determine, for a selected base pair, deviations of hydrogen bonds from ideal Watson–Crick hydrogen bonding (39). Using this method we also calculated the quality of Watson–Crick hydrogen bonding at the adducted site. Deviation from ideal hydrogen bond angles and distances was calculated as:

\begin{equation*} I_H = \sum D - H \ldots A[(dDA - d0DA)2 + (1 + \cos \gamma )2] \end{equation*}

In an ideal C:G bond, distances would be: O6 (G) to N4 (C) = 2.91 Å (bond 1); N1 (G) to N3 (C) = 2.95 Å (bond 2); and N2 (G) to O2 (C) = 2.86 Å (bond 3). The ideal bond angle is always 180°. An ideal result from the I_H_ equation would be 0 and a deviation from this is a change in the ideal structure. Median I_H_ values across simulation time points were calculated at the adducted base pair for each sequence.

### DNA structural parameters measured

Variation for 17 DNA structural parameters was analysed for the sequences in this study to provide a global view of DNA conformational distortion post-BPDE adduction. Six intra-base pair parameters—shear, buckle, stretch, propeller, stagger and opening—collectively define the position and orientation of two bases relative to ideal base pair geometry. Ideal or reference values of these parameters describe canonical Watson–Crick base pairs whereas non-ideal values describe deformations with respect to the short and long axis of the base pairs as well as their normal. Buckle, propeller and opening are rotations whereas shear, stretch and stagger are translations. Six inter-base pair parameters—shift, tilt, slide, roll, rise and twist—define the position and orientation of two consecutive base pairs as a base pair step. Shift, slide and rise are translations whereas roll, tilt and twist rotations. The base pair axis parameters X and Y displacement, as well as axis-bend, inclination and tip, were also measured and describe the geometry of the base pair relative to the helical axis. Each of the parameters can provide an insight into the unique structural deviation of each sequence. MD trajectory files for helical parameter data across base pairs for all sequences over time were generated and visualized using the open source Curves+ and Canal software (40). Helical parameter data for each base pair were compared to published reference values (41).

### Statistical analysis of data distributions

Each data distribution was tested for normality using the Anderson–Darling test. All structural parameter data distributions were non-normal and a Mann-Whitney *U* test used to determine any statistical differences between adducted and control sequence data.

### Multivariate statistical analysis

Multivariate statistical analysis was applied to the output structural data to interpret the distortion caused by adducts relative to the same non-adducted sequence as well as between sequences. Multiple factor analysis (MFA) is an extension of principal components analysis (PCA) designed to analyse multiple data tables that measure sets of variables collected on the same observations (42). In this study, the observations are the different DNA sequences and a variable set represents the measurements across the sequence for a given structural parameter. Thus, in total, there were 17 variable sets representing each structural parameter. A variable set contained either 10 measurements for intra-base pair parameters or 11 measurements for each of the other parameters. MFA elucidates the common structures present in all or some of these sets and the method performed in two steps. Firstly, a PCA was performed individually on each structural variable set that is subsequently normalized by dividing all its elements by the square root of the first eigenvalue obtained by PCA. Secondly, the normalized data sets are combined to form a unique matrix and a further global PCA performed on this matrix. Each of the individual structure variable sets was finally projected onto the secondary analysis to determine the degree of structural differences between adducted and non-adducted sequences. MFA was performed using the ‘mfa’ function from the FactorMineR package (43) in R statistical environment (44).

### Mutability of adduct site sequence context in lung cancer

The mutability of the local sequence contexts at each mutation hotspot and non-hotspot site, was compared by examining the pattern of mutation frequencies (G:C>T:A, G:C>A:T, G:C>C:G, A:T>T:A, A:T>A:G, A:T:C:G) at each adduct site with consideration of both 5′ and 3′ bases (i.e. the effects of neighbouring bases in a trinucleotide sequence). Base substitution data were retrieved for the entire *TP53* gene from the IARC *TP53* database (33) for lung cancer designated smokers or non-smokers with no other known exposure. Using the format of Alexandrov *et al*. (45,46), counts of substitutions across *TP53* represented by C>A, C>G, C>T were made for each of the trinucleotides ACA, ACC, ACG, ACT, CCA, CCC, CCG, CCT, GCA, GCC, GCG, GCT, TCA, TCC, TCG, TCT and for T>A, T>G, T>C at trinucleotides ATA, ATC, ATG, ATT, CTA, CTC, CTG, CTT, GTA, GTC, GTG, GTT, TTA, TTC, TTG, TTT. Thus, all substitutions are referred to by the pyrimidine of the mutated Watson–Crick base pair within trinucleotides yielding 96 possible mutations by sequence. The frequency of each of the 96 mutation types was calculated separately for smokers/ex-smokers and non-smokers to produce a mutation signature for each group. The frequencies of G:C>T:A mutations at sequences for all hotspots and non-hotspots were then compared in each group to determine any sites with increased relative mutation counts.

## RESULTS

Using MD simulations and multivariate statistical analysis we investigated how different *TP53* sequence contexts contributed to variability in DNA distortion at BPDE adduct sites relative to the same non-adducted sequences. Firstly, the conformational stability and flexibility of each sequence was calculated as all atom RMSD values for the entire structure in each case. Secondly, disruption of hydrogen bonding at the adducted base was assessed. Thirdly, helical parameters including intra- and inter-base pair rotational and translational movements as well as base pair axis provided measurements on the type of structural distortion at adduct sites. The overall distortion at each adducted sequence according to these parameters was interpreted using multiple factor analysis.

### Conformational stability and sequence flexibility

**Figure 2.**
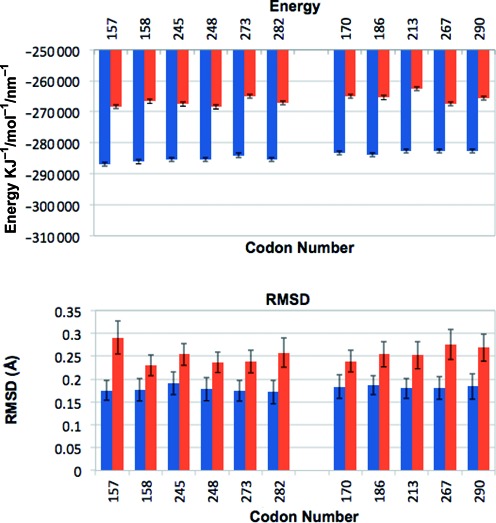
Conformational stability and flexibility of adducted and control sequences. (**A**) Total Energy in KJ/mol for control sequences (blue) and adducted sequences (red); error bars show +/− standard deviation. (**B**) RMSD values for adducted sequences (red) and non-adducted control sequences (blue) taken as an over the simulation time for each sequence.

RMSD analysis and energy level calculations provided confidence about the stability of the simulations. If RMSD values are high then flexibility and movement during a simulation are likely to be unstable. Total energy calculations (KJ/mol) for each sequence showed all simulations to be stable (Figure 2A). RMSD values were calculated as an average for each sequence relative to the starting structure providing an indication of flexibility differences over the 10 ns simulation time (Figure 2B). The adducted sequence had RMSD values of between 0.16 ± 0.02 nm and 0.20 ± 0.03 nm for the first 300 ps of simulation and between 0.23 ± 0.2 nm and 0.29 ± 0.3 nm thereafter. Fluctuations around the mean were stable between 0.3 ns and 10 ns of simulation and it was this time period used all further analysis. Further to this, the RMSD results show a general increase of flexibility in the adducted sequences compared to the non-adducted controls. This is to be expected as a large adduct induced by BPDE will interfere with the helical structure and as such cause more fluctuation and movement. All differences in the distributions of RMSD values between adducted and control sequences were significant (*P* < 0.000).

### Hydrogen bonding quality index

**Figure 3.**
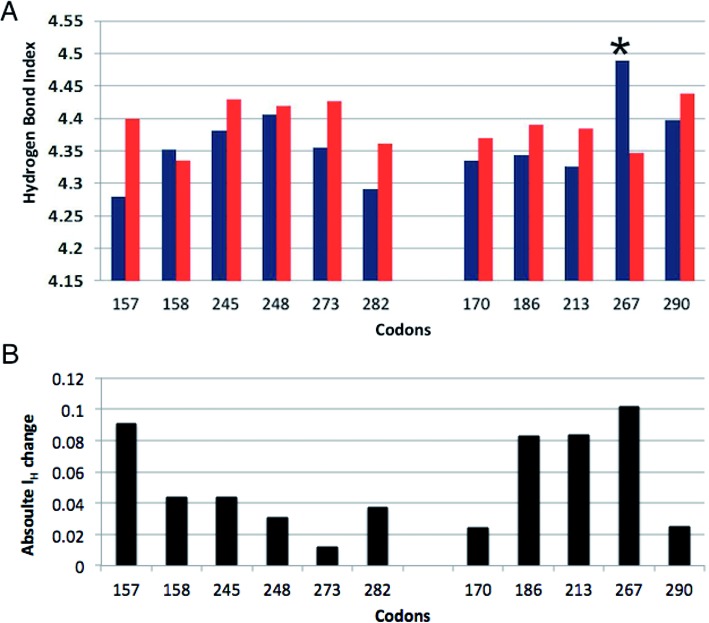
Hydrogen Bond Quality Index (I_H_). (**A**) Median I_H_ scores for the sixth base (guanine) adduct site for adducted sequences (red) and non-adducted control sequences (blue). Error bars represent the inter-quartile range. (**B**) Absolute difference between I_H_ scores for adducted and non-adducted sixth base. * denotes a significant difference between adducted sequence and control (*P* < 0.05).

The deviations from ideal Watson–Crick hydrogen bonding and angles for the sixth base adduct site were quantified for all 11 sequences and their corresponding controls using the hydrogen bond quality index (I_H_). Values for I_H_ (Figure 3A) show some change from ideal structure ranging from 4.50 to 4.64 in controls and 4.51 to 4.65 in adducted sequences. A significant difference for I_H_ was observed for codon 267 (*P* = 0.030) where the median value in adducted sequence was reduced relative to control. For all other sequences apart from codon 158, the median I_H_ increased with an adduct present. The absolute differences at sixth base between I_H_ for adduct and control sequences were generally increased for codons that are uncommon mutation sites in cancer. For all codons, the median I_H_ values, median bond distances and angles along with interquartile range for each parameter are provided in Supplementary Table S1.

### Comparison of structural distortion in *TP53* adducted sequences by multivariate analysis of helical parameters

For each helical parameter, movement medians were calculated from trajectory files over 4851 time frames, spanning from 300 ps to 10 ns of the simulation time. The first 300 ps of real time were disregarded and the remaining time steps (9700 ps) were used to build up a dynamic picture of fluctuations in each structural parameter for every sequence. Median parameter values across each sequence were visualized graphically and assessed for sequences with relative increased distortion.

After inspection, values for the first and last base pairs for all parameters were removed due to high end of sequence fluctuations in movement. The remaining data were collated as 17 variable sets as input to MFA. The normalization process during the first step of MFA ensures that the singular value of the principal component for each parameter variable set is equal to 1. Thus, the normalization step prevents values from any one structural parameter dominating the MFA solution and separation of DNA sequences.

**Figure 4.**
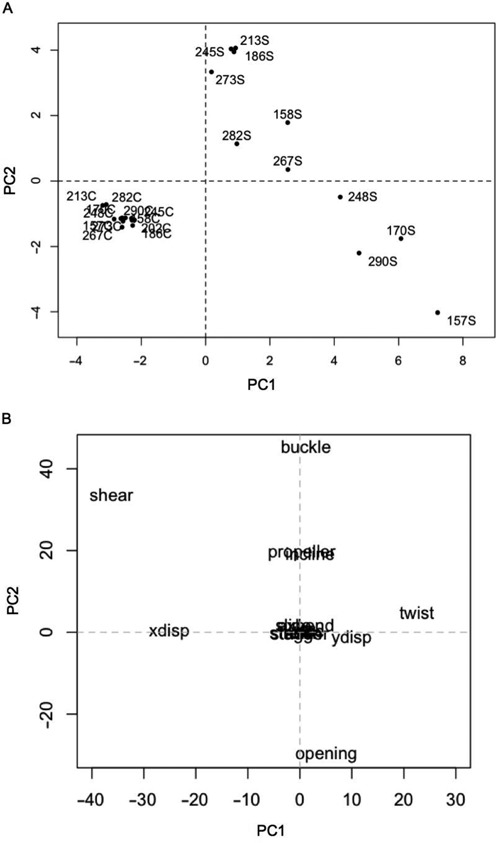
Multiple factor analysis of adducted and control DNA sequences. (**A**) The scatterplot shows how all adducted and control sequences correlate with the first component (PC1) and the second component (PC2). Sequence labels end in ‘S’ to denote the 10S BPDE adduct whereas control sequence labels end in ‘C’. (**B**) The scatterplot shows how each of the structural parameters correlate with PC1 and PC2.

The first two components (PC1 and PC2) from the global MFA solution explained 41.12% and 20.91% of the data set variation, respectively. This solution provides a map of the similarities between sequences for distortion levels and an overall insight into the interplay between the structural parameters that cause this distortion. A scatterplot of PC1 and PC2 shows how sequences correlate with each component (Figure 4A). PC1 conclusively separates all adduct from control sequences. Furthermore, sequences with adducted codons 157, 170, 248 and 290 have the highest correlation with this dimension and greatest structural differences from controls. PC2 further separates these four sequences from the other adduct sequences with each having a negative correlation on the component. There is very little variation for distortion between non-adducted sequences.

The contribution of each structural parameter to the variation observed between sequences was determined by examining the correlation of each of the parameters on PC1 and PC2 (Figure 4B). Shear and x-displacement had an increased negative correlation on PC1 and are thus deemed to contribute strongly to the structural distortion caused by the presence of a BPDE adduct in each sequence. The twist parameter also contributes to this distortion. Buckle and opening have the highest correlation with PC2 and thus have the greatest influence on differing types of structural distortion between adducted sequences.

### Characterization of DNA BPDE-induced distortion at *TP53* codon 157

**Figure 5.**
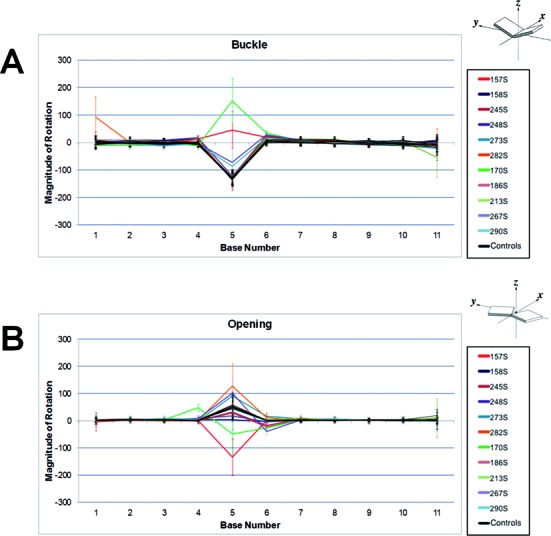
Line graph representations of median magnitude of rotation values for every base across each adducted and non-adducted control sequence. (**A**) Median buckle angle. (**B**) Median opening angle. Control sequences are black. Values are shown for every base across each adducted and non-adducted control sequence. Control sequences are black. Error bars signifying the interquartile range are provided for each base per sequence. The diagrams representing buckle and opening are reprinted with permission from Lu and Olson (47). Copyright 2003 Oxford University Press.

The line graphs shown in Figure 5 (47) represent the median magnitude of rotation for buckle (Figure 5A) and opening (Figure 5B) per base. Line graphs for all other parameters are shown in Supplementary Figure S1. Buckle and opening are intra-rotational base pair parameters describing rotation around the x and z base pair axes. The buckle rotation is increased in all adducted and control sequences at the fifth base corresponding to the methylated cytosine in the central CpG site and adjacent to the adducted site. With the exception of codon 157 and codon 170 sequences, the direction of buckle is uniform in all other adducted sequences as well as all control sequences, each having similar levels of median distortion.

The opening parameter also shows uniformity for magnitude of rotation at most bases in the control sequences, but an increase in magnitude of rotation at the methylated fifth base. The magnitude of rotation was increased in some adducted sequences relative to controls but decreased in others. Once again, the direction of opening angle in the adjacent methylated cytosine of codons 157 and codon 170 is opposite to the angle in the same control sequences as well as all other sequences, adducted or not. A slight increase in magnitude of rotation was observed for some other adducted sequences at the sixth base.

**Figure 6.**
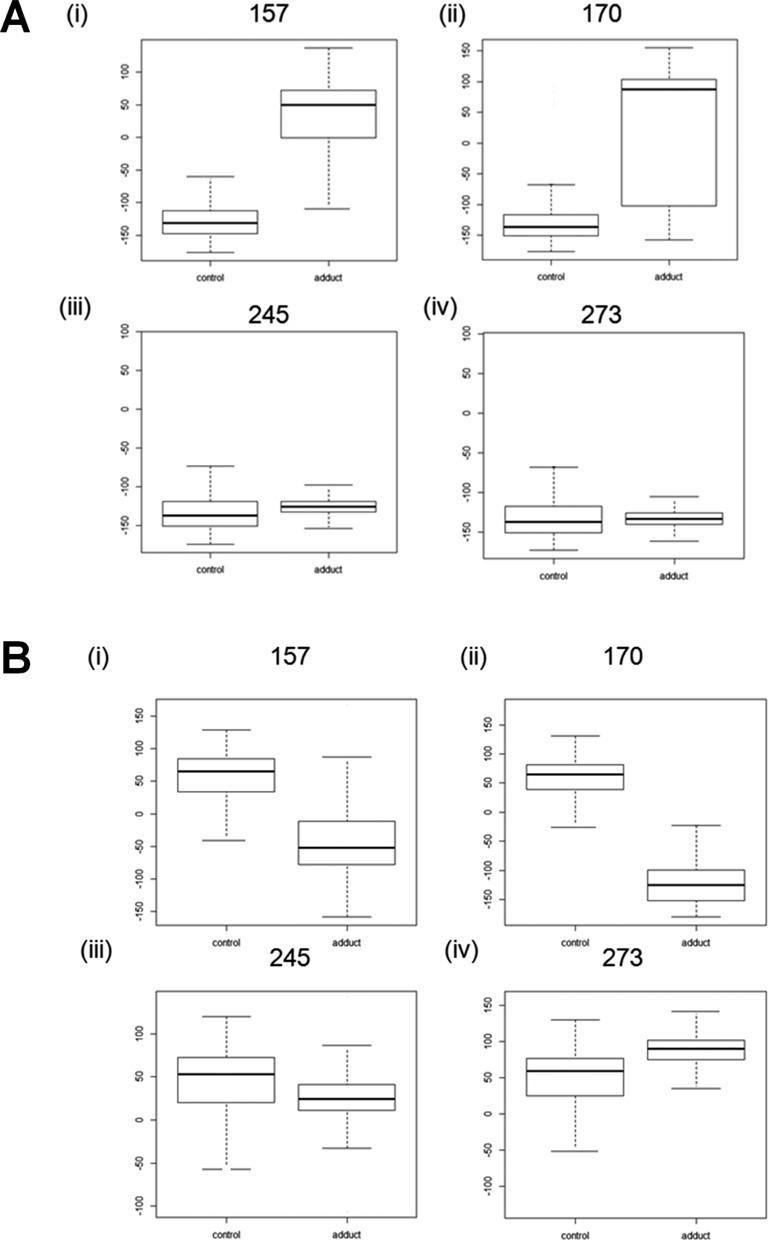
Boxplots for the magnitude of rotation at the fifth base in selected adducted and control sequences. (**A**) buckle at: (i) codon 157; (ii) codon 170; (iii) codon 245; (iv) codon 273. (**B**) opening (i) codon 157; (ii) codon 170; (iii) codon 245; (iv) codon 273. Each plot shows the median, quartile, maximum and minimum values.

We compared the distributions of magnitude of rotation values over time (i.e. all values over 10 ns) for buckle and opening at the fifth base in codon 157 with mutation hotspot codons: (i) 245 and 273, that grouped distally after MFA; (ii) 170 that grouped more closely (Figure 6). The rotation distributions for adducted codon compared to control were significantly different in each case (*P* < 0.0001). The range of buckle rotation values for codon 157 with adduct present (median = 49.51) over time had very little overlap with control values (median = −131.08) highlighting limited fluctuation (Figure 6A). Adduct-induced buckle in codon 170 (median = 87.27) showed a larger distribution relative to control (median = −136.35) indicating a higher degree of fluctuation in magnitude of rotation. These results contrasted with the overlapping time-dependent buckle distributions observed for codons 245 and 273. The distribution patterns in magnitude of rotation for opening followed a similar pattern to buckle. The range in magnitude of adduct-induced opening rotation for 170 (median = −51.62) however showed little overlap with the control distribution (median = 65.55) (Figure 6B). We observed that the distributions for buckle and opening for codon 248 were similar to that for codon 245 (Supplementary Figure S2). This confirmed that the rotation values for codon 248 was dissimilar to codons 157, 170 and 290 as dictated by MFA.

**Figure 7.**
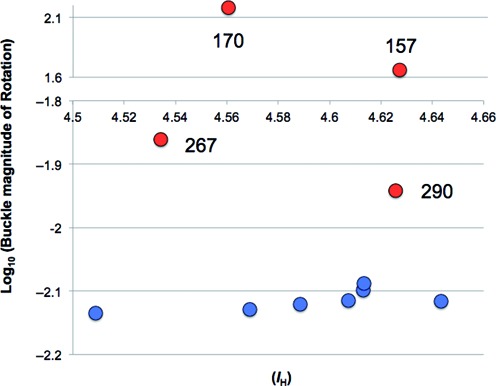
Plot of median hydrogen bond quality index data (*I*_H_) and Log_10_ of values for buckle magnitude of Rotation. Log values for codons with a negative value for buckle have negative signage. The y axis is cut between −1.8 and 1.6 to aid visualization.

Although not significant (*P* = 0.052), the degree of difference for the hydrogen bond quality index between codon 157 adducted (I_H_ = 4.63) and control (I_H_ = 4.54) sequences was increased relative to most other codons (Figure 3). This contrasts with codon 267, which had a reduction in I_H_ suggesting lower disturbance to the base-pair parameters. Any relationship between I_H_ at the adducted base and the magnitude of buckle at the methylated cytosine was explored by plotting these values against each other (Figure 7). With the exception of codons 170, 267, 157 and 290, there is a subtle linear increase in the Log10 values of buckle as I_H_ increases across codons. No such patterns were observed for opening (data not shown).

### Mutability of adduct site sequence contexts in lung cancer

**Figure 8.**
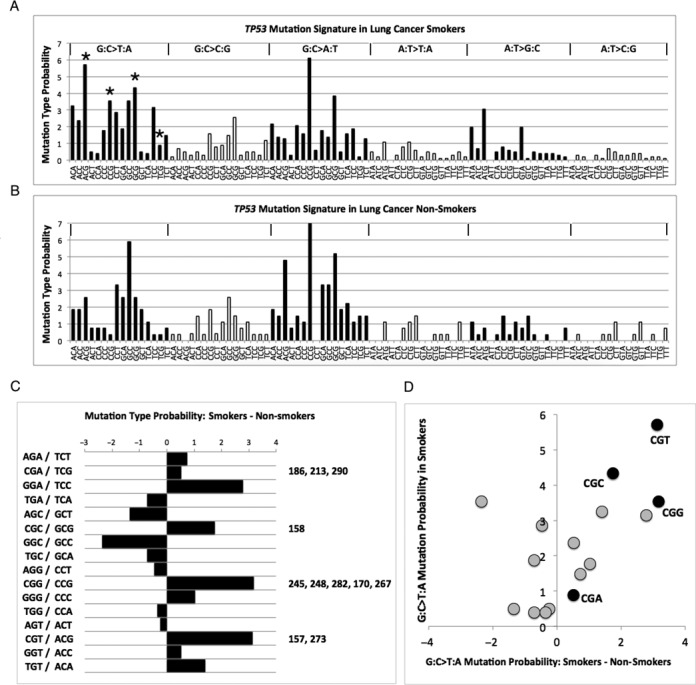
*TP53* Mutation signatures for lung cancer. (**A**) Mutation signature for smokers showing the probability for different mutation types at relevant trinucleotides. The trinucleotide sequences used in the study are marked with an asterisk. (**B**) Mutation signatures for non-smokers. (**C**) Difference between smokers and non-smokers for G:C>T:A mutation probabilities. Trinucleotide sequences used in this study are labelled. (**D**) Plot of G:C>T:A probabilities at relevant trinucleotides in smokers against probability differences between smokers and non-smokers.

To provide insight into how structural distortion might associate with mutation frequency, we assessed mutability of local sequence context at each adduct site. Mutation signatures for the entire *TP53* gene were generated for smokers and non-smokers and mutation type probabilities at trinucleotides common to the adduct sites studied by MD were compared. The lung cancer mutation signatures for smokers and non-smokers are shown in Figure 8. The patterns for G:C>T:A mutations between smoker (Figure 8A) and non-smoker (Figure 8B) clearly contrast. The probability for G:C>T:A mutations in smokers (5.71) was highest at CGT/ACG trinucleotides as found encompassing mutable guanines in codons 157 and 273. The second highest mutation probability (4.33) was observed at CGC/GCG (codon 158) trinucleotides followed by CGA/TCG (3.54) and reduced further for TCG/CGA (0.89). We then subtracted the G:C>T:A mutation type probabilities for non-smokers from smokers (Figure 8C) to reveal the relative smoking-related increase in mutability for all trinucleotides used in this study. A similar mutation probability increase was observed for CGT/ACG (3.13) and CGC/GCG (3.17) sequences. Lower probability increases were observed for CGC/GCG (1.75) and TCG/CGA (0.52).

The probability of G:C>T:A mutations in smokers for each of the trinucleotide types was plotted against the probability differences between smokers and non-smokers (Figure 8D). An interesting trend was observed. Across trinucleotides, there was a significant correlation (0.580, *P* = 0.018) between the probabilities of a G:C>T:A mutation occurring in smokers and the magnitude of differences between the probabilities of G:C>T:A mutations in smokers relative to non-smokers. In other words, for a trinucleotide, if there was an increase in the probability of a G:C>T:A mutation in smokers this did not mean that the probability was increased in non-smokers and vice versa. This overall effect was greatest for the CGT/ACG trinucleotide. This trinucleotide contains the sequence context for the adducted guanine with 5′ cytosine and 3′ thymine at mutation hotspot sites in codons 157 and 273. Analysis of available data from the IARC *TP53* database showed that over 93.1% (27/29) of all substitutions at the adducted guanine in codon 157 are G:C>T:A where the smoking status was known. Of these mutations, 96.3% (26/27) were observed in smokers. Conversely, G:C>T:A make up 62.1% (36/58) of substitutions at the adducted guanine in codon 273. Of these, 83.3% (30/36) were observed in smokers. Consequently, G:C>T:A mutations in smokers make up 89.7% (26/29) of all substitutions observed at the guanine in codon 157. This contrasts with G:C>T:A mutations in smokers which contribute to 51.7% (30/58) of substitutions in codon 273.

## DISCUSSION

It is known that local 3D structure and flexibility of the DNA helix are dependent upon the sequence of bases in the helix (48). Many studies have attempted to determine the rules of this sequence dependence, and how sequences impact upon DNA replication, repair and mutation rates (49,50). Previous molecular modelling studies have focused on the ability of BPDE to bind to guanine (48–52). Further to this, curvature and flexibility of DNA have previously been studied using MD methods (51–53) and many studies have been carried out into the effects local sequence has on damaged base conformation (18), influence of mutagenic potency (16,54–55), as well as on the effects of bending DNA (56,57). There have also been studies into the influence neighbouring bases have on the likelihood of a mutation at a site including adjacent as well as more distal bases (28).

Base sequence context has been shown, *in silico*, to affect the process of nucleotide excision repair (NER) (29,30) due to slow repair (19) in addition to sequence dependent structural distortion (36). The rate by which the NER repair mechanism excises different bulky adduct lesions such as BPDE in different sequence contexts varies hugely (22–25,58). Many studies, both experimental and theoretical, show the importance of distortions such as kinks, distortions in hydrogen-bonding and flipped nucleotides. The specific way in which these changes affect structure and mutation/repair is unclear (50,59–64).

In the NER pathway, it is the heterodimeric XPC-RAD23B protein which recognizes the local distortion or destabilization of the DNA that has been caused by a lesion or adduct. It then binds to this damage site causing the DNA strands to partially separate before further separation and replacement of nucleotides. A recent structure of the yeast recognition factor has been crystallized and Cai *et al.*, suggest that this structure points towards local thermodynamic stability at the site of the adduct playing an important role in whether the region shows a high or slow rate of repair, relating to the particular adduct and sequence (31). Mu *et al.*, in comparing NER assay results with structural information obtained from MD simulations, highlight variation in stacking stabilization between different types of adducts and different sequence contexts (65). Another study by Donny-Clark *et al.*, explored the association of sequence effects upon BPDE adducts and showed correlation with known NER efficiencies. Thus, it is important to assess variance and type of structural distortion due to sequence context when trying to elucidate the repair rate at mutation hotspot sites (31,66–68).

This study expands on the use of molecular modelling to elucidate the effects of DNA sequence context on structural distortion caused by BPDE in the *TP53* tumour suppressor gene. The 17 helical parameters analysed for the *TP53* hotspot sites can be used to describe structural changes in the DNA helix. The use of multivariate statistics to explore adduct-induced structural distortion from MD simulations at mutation hotspot sites in a tumour gene has not been demonstrated previously.

The eleven 11 bp mutation sequences chosen all had a central CpG site at bases five and six. Thus, distal or adjacent 5′ or 3′ bases must influence any BPDE adduct-induced differences in structural distortion observed due to sequence context. A large number of mutations for BPDE, particularly G to T transversions, are observed at methylated CpG sites. One hypothesis as to the increased frequency of BPDE adducts at CpG sites is that the hydrophobicity of the methyl group in the methylated cytosine enhances adduct formation at the guanine base (26). Sequence context then influences the subsequent distortion effects adducts can have on the DNA as well as the ability of the DNA repair enzymes to remove the mutagen and correct the mutation (29,30).

A global analysis of the parameter data across sequences by MFA was able to provide an indication of the overall degree of adduct-induced structural distortion in each case. MFA determined that overall distortion patterns were different in sequences with adducts at codons 157, 170 and 290. According to the IARC *TP53* Database (version R17) (33) over 30% of G to T transversions, a hallmark of BPDE adduct formation, observed at codon 157 in cancer were in lung tumours. Thus, codon 157 is deemed a lung cancer specific mutation hotspot in smokers. Conversely, G–T transversions at codon 170 are silent and at codon 290 result in a substitution of arginine–leucine. Both these mutations are rarely observed in cancer. That said, when these mutations do arise in codon 170 they are most common in lung tumours. It is also worth noting that whereas BPDE has a strong binding affinity for codon 157, the affinity is weaker for both codon 170 and 290 (8). The sequence with codon 248 did also separate in MFA but had a low correlation on PC2 and was thus not considered further.

MFA revealed that the buckle and opening parameters contributed strongly to the structural differentiation of the adducted sequences. The buckle parameter is linked to the positioning of the bases and sugars in the nucleotides. The breaking or disturbing of the hydrogen bonds has a big impact on the magnitude of buckle when the bases move out of position and shorten or lengthen the distance between the pair. The buckling caused by the BPDE adduct moves the base pairs from pointing towards the centre of the helix to point towards either the 5′ or 3′ direction. The direction of adduct-induced buckle at the fifth base in codons 157 and 170 was towards the 5′ base. The direction of buckle in all other sequences was towards the 3′ base. Opening is also a rotational parameter and measures the rotation of the bases about an axis perpendicular to the plane of the base pair. Opening measures a compression of the major or minor groove and in this case indicates a movement at the fifth base to accommodate the large bulky adduct. Unlike buckle, the direction of adduct-induced opening at the fifth base in codons 157 and 170 was towards the 3′ base whereas direction was towards the 5′ base in all other sequences. This suggests, given the variability across all sequences for bases either 3′ or 5′ to the CpG sites, that the neighbouring base alone does not influence the direction of buckle or opening.

Hydrogen bond quality index data (*I*_H_) for each adducted base showed an interesting pattern. The ideal Watson–Crick hydrogen bonds have an *I*_H_ value of 0. The value of *I*_H_ increases as the hydrogen bond distances or angles diverge from ideal Watson–Crick hydrogen bond values. Hydrogen bond distance and angle deviations are produced by different combinations of distortions due to a number of structural parameters including buckle and opening. On first inspection, the pattern of adduct-induced changes in *I*_H_ values for codons was not reflected by the magnitude of rotation patterns for either buckle or opening. However, if one ignores buckle at codons 170 and 267 there is an apparent non-linear association between increased disruptions of hydrogen bonding and increasing magnitude of buckle.

The relative changes in median *I*_H_ values suggest that local hydrogen bond disruption is dependent on more distal neighbouring bases than just those immediately 5′ or 3′. The trinucleotide sequence (CGT/ACG) of codons 157 and 273 are the same surrounding the adducted guanine. Similarly, codon 267 shares a CGG/CCG sequence with 170, 245, 248 and 282. There is evidence that more distal bases effect the disruption of hydrogen bonding at the damaged base. Cai *et al*., used molecular dynamics simulations to study the effects distant base neighbours have on the ability of NER to repair 10S (+)-trans-anti-B[a]P-N(2)-dG minor groove lesions in two sequences that had the same adjacent bases to the adducted guanine (CGC/GCG) but different distal bases (28,30). They observed that, despite the same neighbouring bases, one sequence was more bent than the other with more torsional flexibility and that these distortions were signals for NER. They identified that the greater bending was due to the amino group of a guanine opposite a cytosine three bases away 3′ acting as a wedge to open the minor groove. This allowed the hydrophobic surface of the BPDE ring system to bury deeper within the groove walls coupled with more untwisting. Interestingly, using unrelated *in silico* analyses, we have previously predicted that the sequence surrounding codon 157 is less flexible than other mutation hotspot sequences (69).

Although not significant compared to control, the increase in *I*_H_ at codon 157 was much greater relative to other sequences. The median bond distances for guanine O6 and cytosine N4 (bond 1) and guanine N1 and cytosine N3 (bond 2) at the adduced base were greater for codon 157 than all other codons (Supplementary Table S1). Thus, it is possible that whereas codons 157 and 273 might both be targeted by BPDE at a high rate there is compromised NER at 157 due to increased structural distortion surrounding the lesion and reduced flexibility across the wider sequence. Further analyses could confirm this.

The significant decrease in *I*_H_ value at codon 267 contrasts with the increase observed for all other sequences with the exception of codon 158. For codon 267, the degree of twist between the C5 and G6 bases and stretch at C5 in codon 267 was increased relative to that for other sequences with the same neighbouring bases (Supplementary Figure S1). Again, this supports the concept that the degree of adduct-induced hydrogen bond disruption is dependent on distal bases. In this case though, the increased local perturbations caused by distal sequence seemingly have the effect of causing the hydrogen bond distances and angles at the adducted guanine to be more similar to ideal Watson–Crick values. Another interesting observation was that non-mutation hotspot sites generally have larger absolute differences between *I*_H_ values for the adducted and non-adducted guanine. This might suggest that disruption to hydrogen bonding is key to damage recognition. We are cautious to make a prediction for BPDE adducts though, as a number of non-mutation hotspot sites analysed are weak binding (e.g. codons 170 and 267). Furthermore, the absolute *I*_H_ difference at codon 157 had a similar level of increase due to adduct formation. This might suggest that, even if hydrogen bond disruption is recognized, the repair process could be compromised depending on the type and position of distortion in the sequence adjacent to the adducted base.

Analysis of *TP53* lung cancer mutation signatures revealed that the G:C>T:A mutability of the trinucleotides analysed in this study are increased by varying degrees in smokers relative to non-smokers. By comparing probabilities for mutation at each sequence and mutability differences between smokers and non-smokers we were able to determine that the CGT/ACG sequence is most mutable for G:C>T:A substitutions in lung cancer. An interesting finding was that, across trinucleotides, the increase in the probability of a G:C>T:A mutation in smokers correlated with an increase in the difference between this probability and that for non-smokers. If there is a greater chance of observing G:C>T:A mutations in smokers at certain sequences that does not mean the chance is also increased in smokers. This finding supports the theory that the aetiology of *TP53* G:C>T:A mutations at certain mutation hotspots in smokers is different to that in non-smokers. The total contributions of adduct-induced damage to different mutation hotspots is however more subtle. The CGT/ACG sequence is shared by the lung cancer mutation hotspots at codons 157 and 273. Further analysis of known smoking related mutation frequencies at these two sites showed, however, that mutations at 157 are almost always likely to be G:C>T:A substitutions and occurring in smokers. This contrasted with codon 273 where other substitution types occur and G:C>T:A substitutions also occur in non-smokers. It is possible that a single mutagen type causes G:C>T:A mutations at codon 157 in smokers but that mutations at codon 273, a hotspot in other cancers, are caused by multiple mutagen types. Combining this knowledge with the MFA results we can suggest that whereas BPDE binds both the 157 and 273 hotspot sites, G:C>T:A substitutions occur at a higher rate at codon 157 in smokers due to structural distortion-induced repair inefficiency. Although empirical data for NER efficiency is lacking for BPDE adducts at CGT/ACG, the reported efficiency of repair is greater at CGG/CCG sites relative to CGC/GCG due to dynamic episodic denaturation of Watson–Crick base pairing flanking the lesion and enhanced recognition (30). This might explain the higher mutation probability of CGG/CCG sites observed in smokers.

The combination of modelled structural distortion data and the *TP53* mutation signatures observed for lung cancer are consistent with codon 157 being a mutation hotspot caused by BPDE adducts in smokers. From our data, we hypothesize that mutation hotspots in lung cancer of smokers arise due to multiple mutagen targeting but that the increased mutation frequency at codon 157 results due to a high level of BPDE binding and reduced NER capacity due to increased structural distortion. One further important observation was that it was clear that the 5′ and 3′ bases at a methylated CpG site alone do not dictate the structural distortion observed when BPDE adducts form. If the repair rate of these bulky lesions is dependent on the degree of structural distortion, then the probability of a G:C>T:A mutation is not just dependent on the local di- or trinucleotide. Thus, although summarizing mutation data at the trinucleotide level is useful, when generating mutation signatures for genes, chromosomes or entire genomes, the effects of more distal bases must also be considered.

As the availability of complete cancer genomes rapidly increases, the wealth of specific mutation data they provide will permit comprehensive analysis of sequence context at mutational hotspots in many genes. Currently, the *TP53* gene is quite unique in that its extensive study has led to the generation of large, curated mutation databases such as the IARC *TP53* database (33) as well as the UMD *TP53* mutation database (5). Thus, the *TP53* gene provides the best model to explore the differential sequence context effects in mutational patterns. Analysis of local sequence context by itself will not be enough to determine the origins of mutations and ultimately the aetiology of the disease. With high performance computing and the speed at which MD can now be performed, molecular modelling of DNA adducts at mutation sites could permit large-scale analysis of sequence dependent local DNA structure. Elucidation of mutagen-induced structural distortion at mutation sites can help explain the mechanism of differential DNA repair within genes. Most importantly, molecular modelling of adducts at cancer gene hotspot sites could help determine the aetiology of mutations in different cancer types. In the same way, this approach could be used to screen mutation hotspot sites for the mutational potential of new and existing drug compounds.

## Supplementary Material

SUPPLEMENTARY DATA
