# Supplementary material for: Base damage, local sequence context and TP53 mutation hotspots: a molecular dynamics study of benzo[a]pyrene induced DNA distortion and mutability
Source: Nucleic Acids Res. 2015 Sep 22;43(19):9133–46. doi: 10.1093/nar/gkv910 (PMC4627081; doi:10.1093/nar/gkv910)
Supplement: SUPPLEMENTARY DATA [file supp_43_19_9133__index.html]

Base damage, local sequence context and TP53 mutation hotspots: a molecular dynamics study of benzo[a]pyrene induced DNA distortion and mutability — Base damage, local sequence context and TP53 mutation hotspots: a molecular dynamics study of benzo[a]pyrene induced DNA distortion and mutability — SUPPLEMENTARY DATA 

# Base damage, local sequence context and *TP53* mutation hotspots: a molecular dynamics study of benzo[a]pyrene induced DNA distortion and mutability

## SUPPLEMENTARY DATA

- SUPPLEMENTARY DATA
- SUPPLEMENTARY DATA
- SUPPLEMENTARY DATA
- SUPPLEMENTARY DATA
