# Supplementary material for: Base damage, local sequence context and TP53 mutation hotspots: a molecular dynamics study of benzo[a]pyrene induced DNA distortion and mutability
Source: Nucleic Acids Res. 2015 Sep 22;43(19):9133–46. doi: 10.1093/nar/gkv910 (PMC4627081; doi:10.1093/nar/gkv910)
Supplement: SUPPLEMENTARY DATA [file supp_gkv910_nar-01475-f-2015-File010.pdf]

SUPPLEMENTARY LEGENDS

Supplementary Table 1 - Median and interquartile range values for IH scores, H-bond distances and H-bond angles at the adduct site guanine within each sequence. Data are provided for adduct and control sequences. See Materials and Methods for description of bonds 1, 2 and 3

Supplementary Figure 1 - Line graph representations of median magnitude of rotation values for all structural parameters analysed (except buckle and opening, shown in Figure 5). Values are shown for every base across each adducted and non-adducted control sequence. Control sequences are black. Error bars signifying the interquartile range are provided for each base per sequence.

Supplementary Figure 2 - Boxplots for the magnitude of rotation for buckle and opening at the fifth base in codon 248 (A) buckle (B) opening (i) codon 157. Each plot shows the median, quartile, maximum and minimum values for adducted and control sequences.
