# Supplementary figures and images for: Base damage, local sequence context and TP53 mutation hotspots: a molecular dynamics study of benzo[a]pyrene induced DNA distortion and mutability
Source: Nucleic Acids Res. 2015 Sep 22;43(19):9133–46. doi: 10.1093/nar/gkv910 (PMC4627081; doi:10.1093/nar/gkv910)

Supplementary Figure 1.

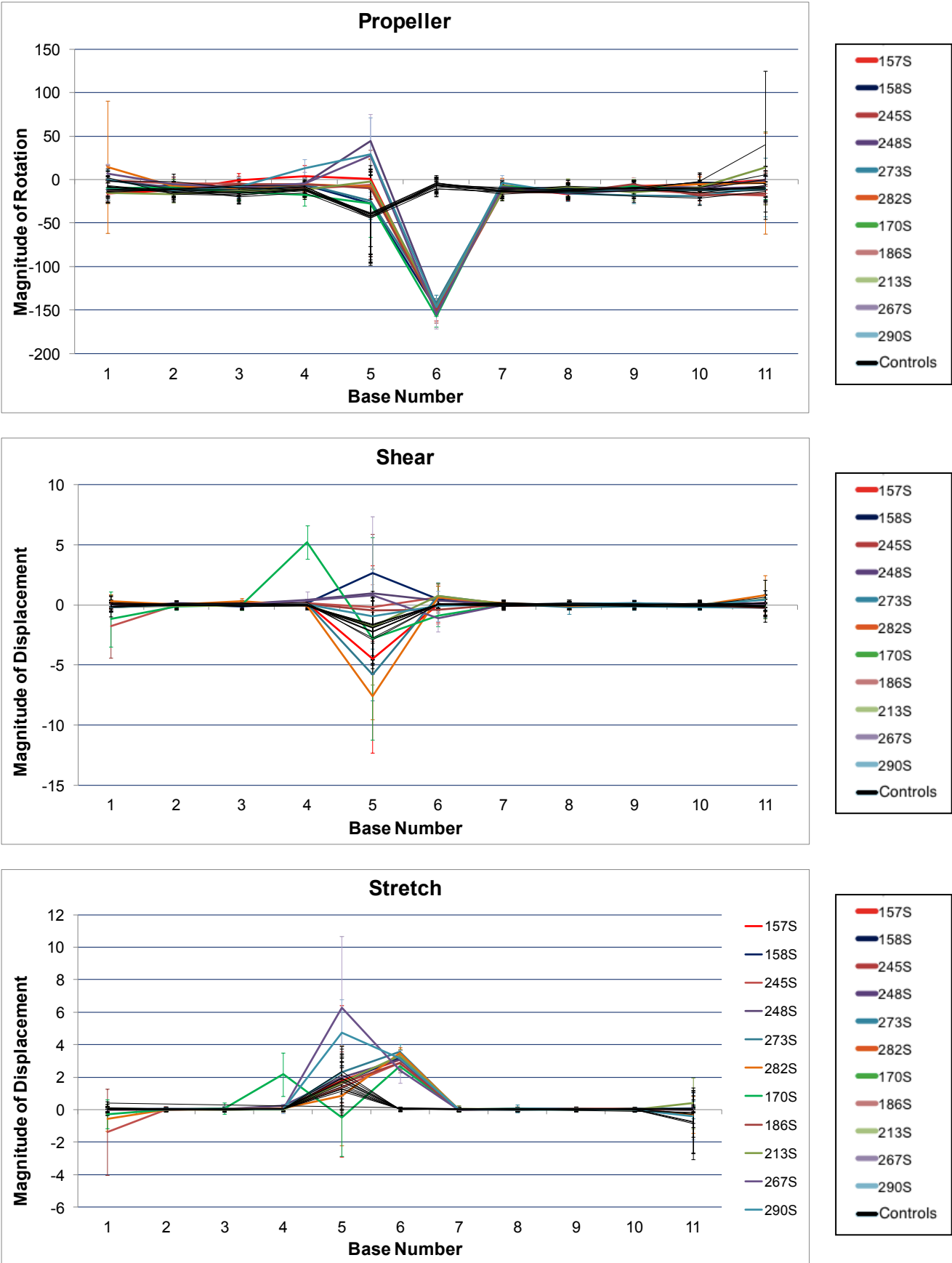

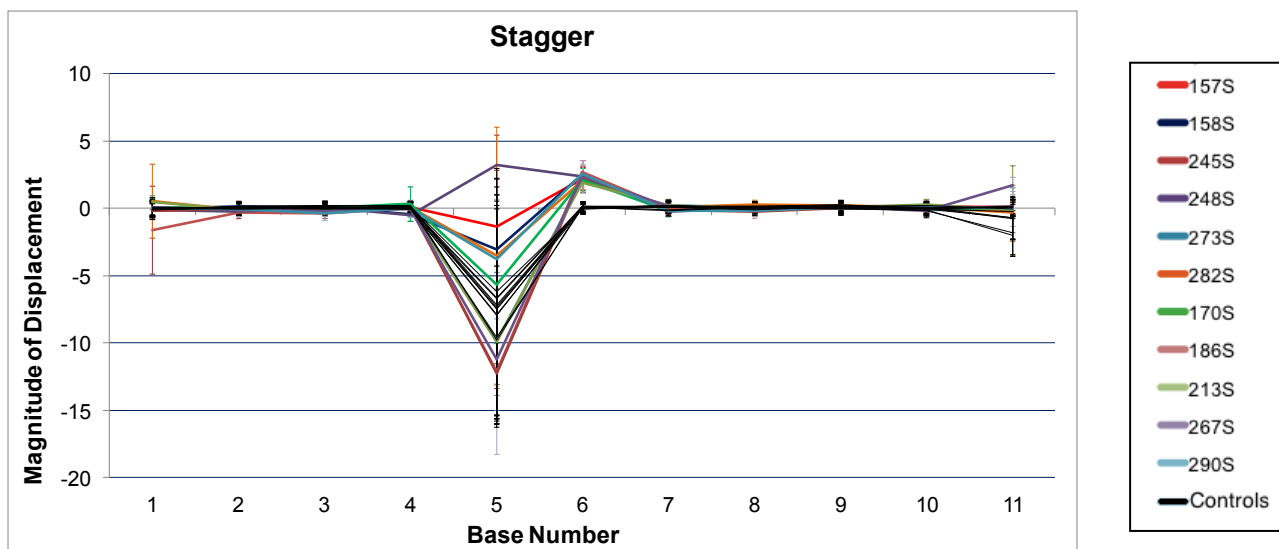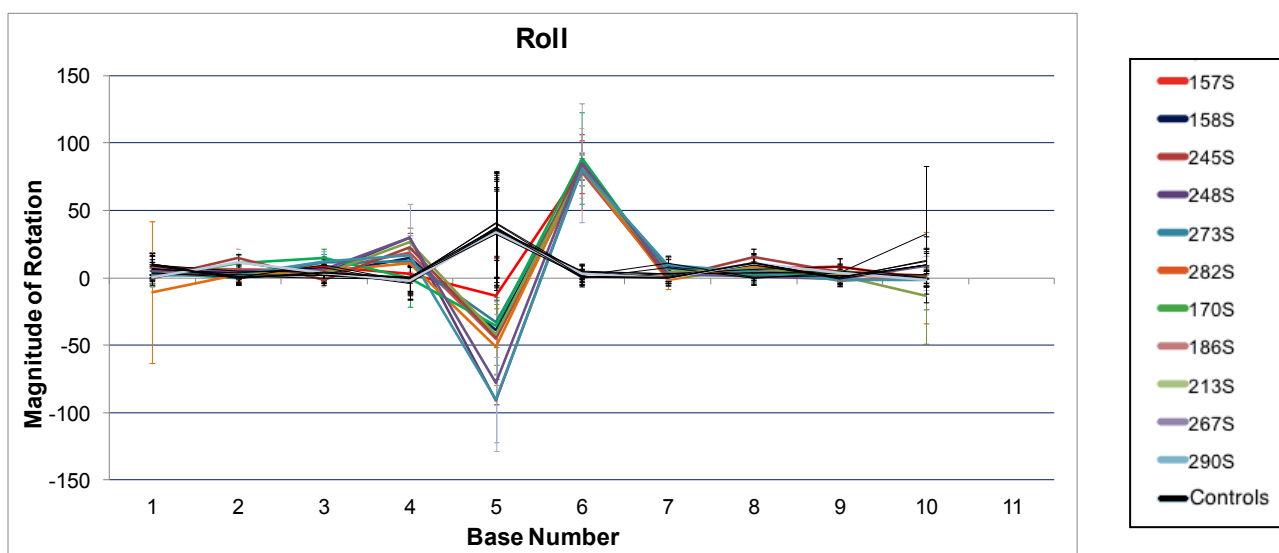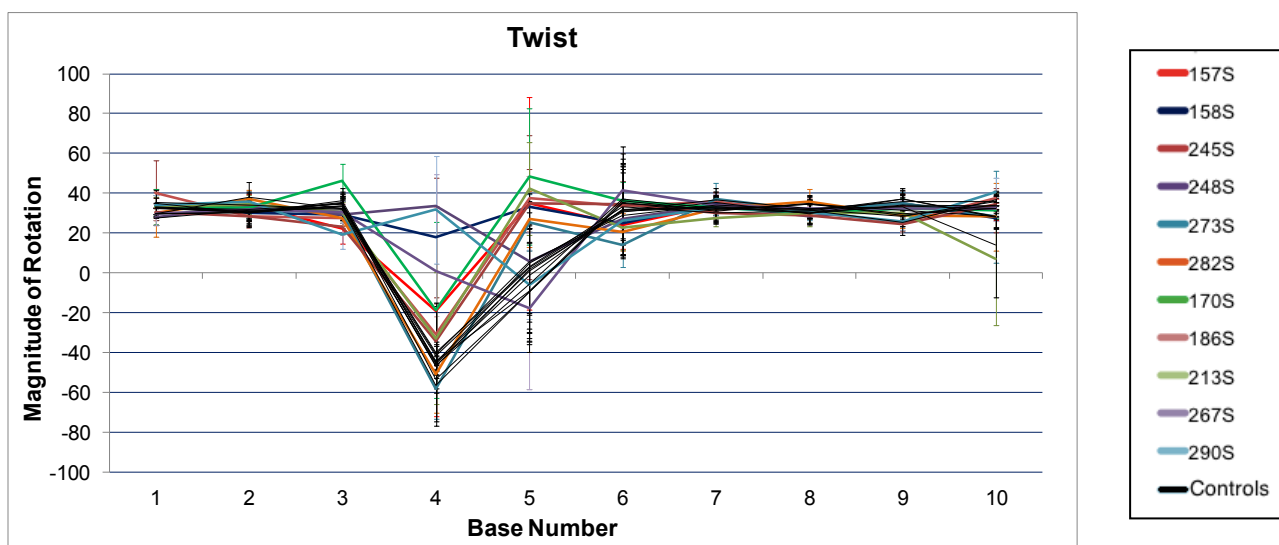



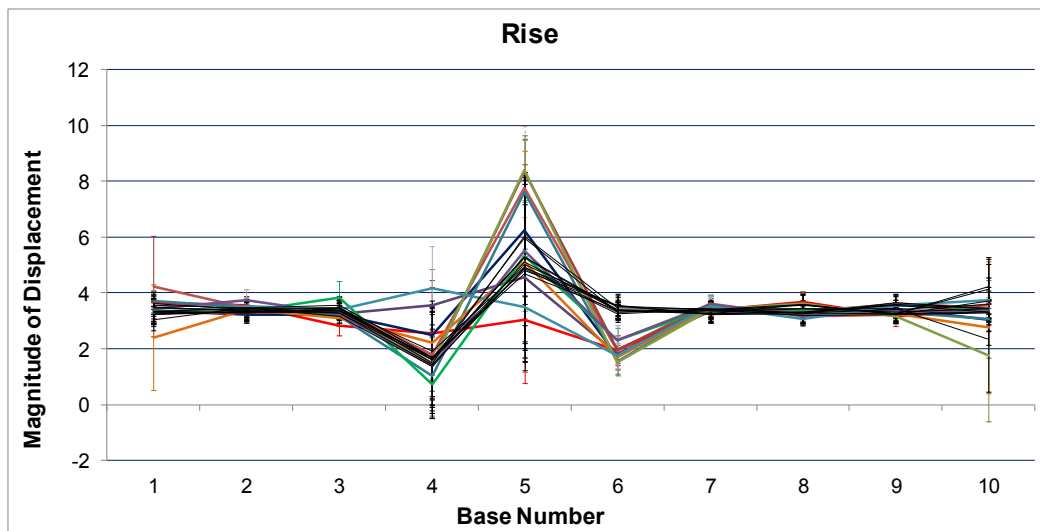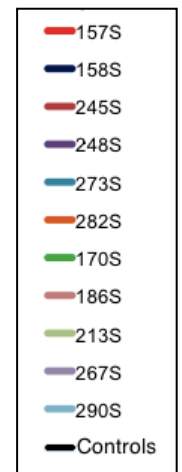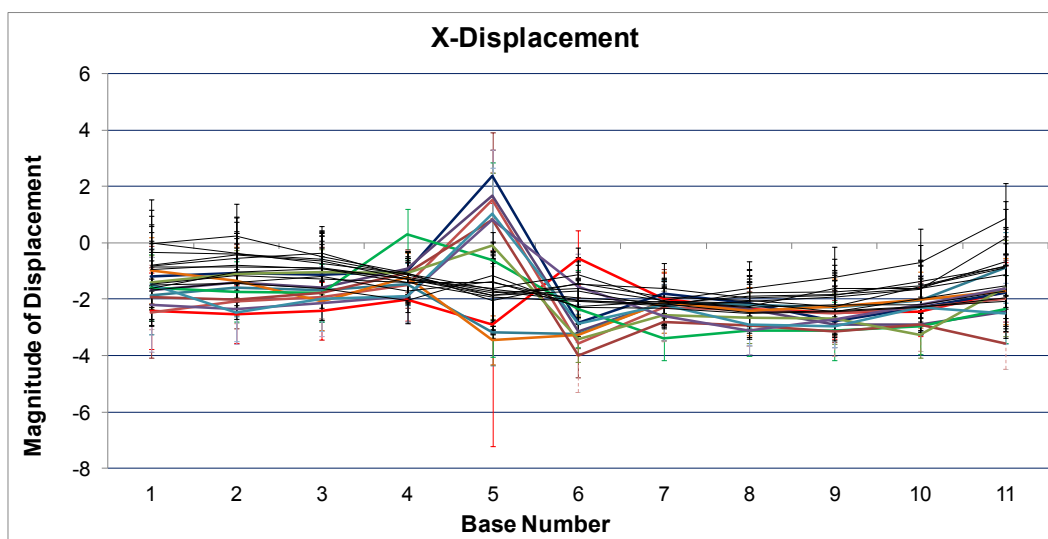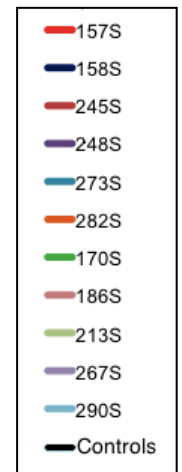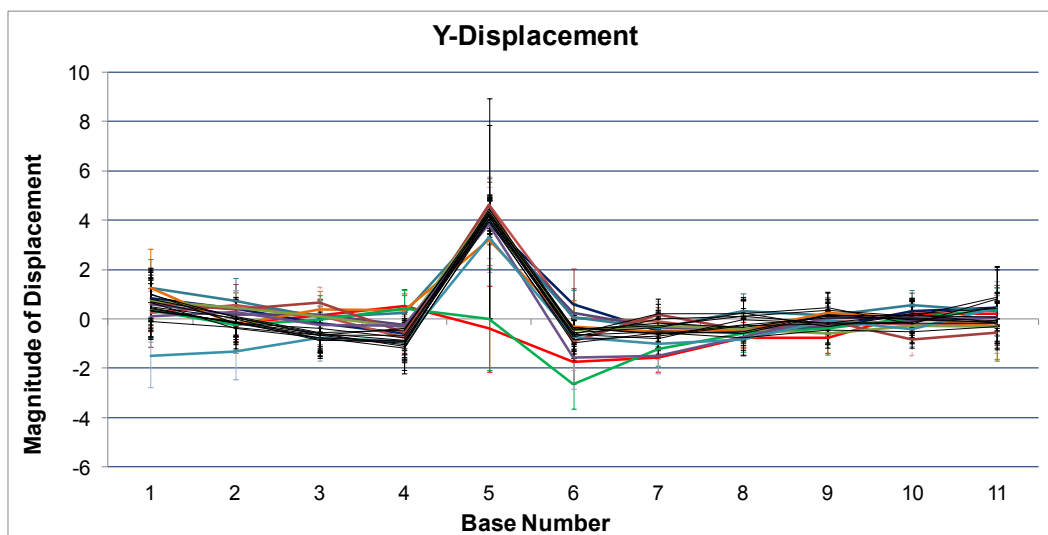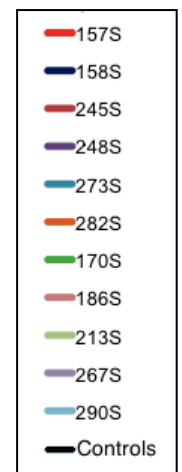

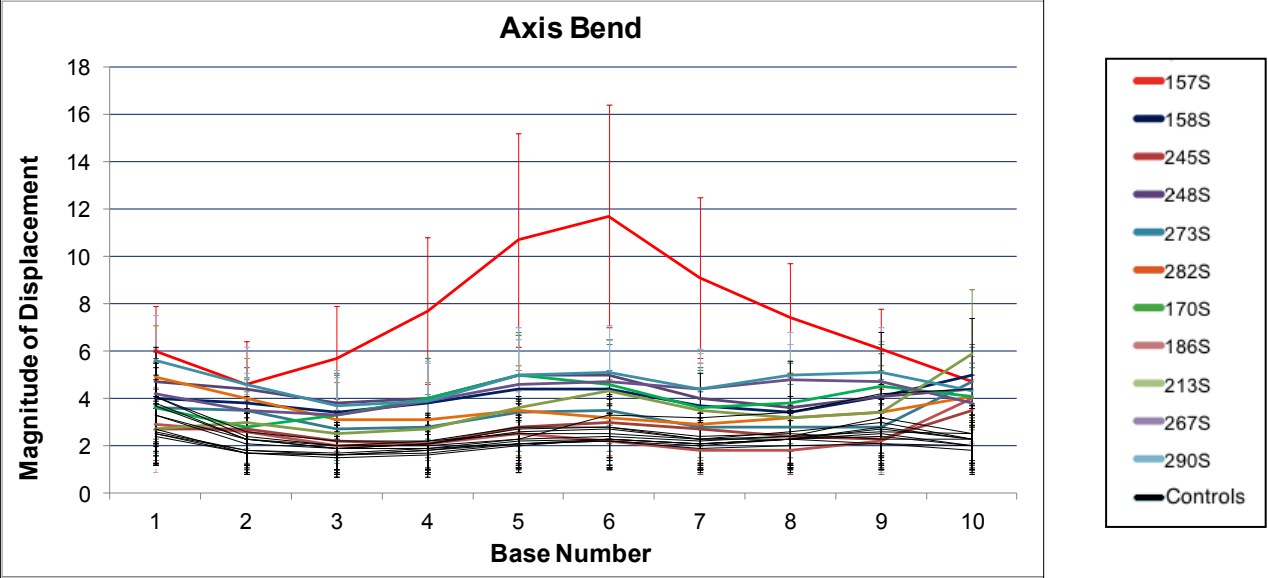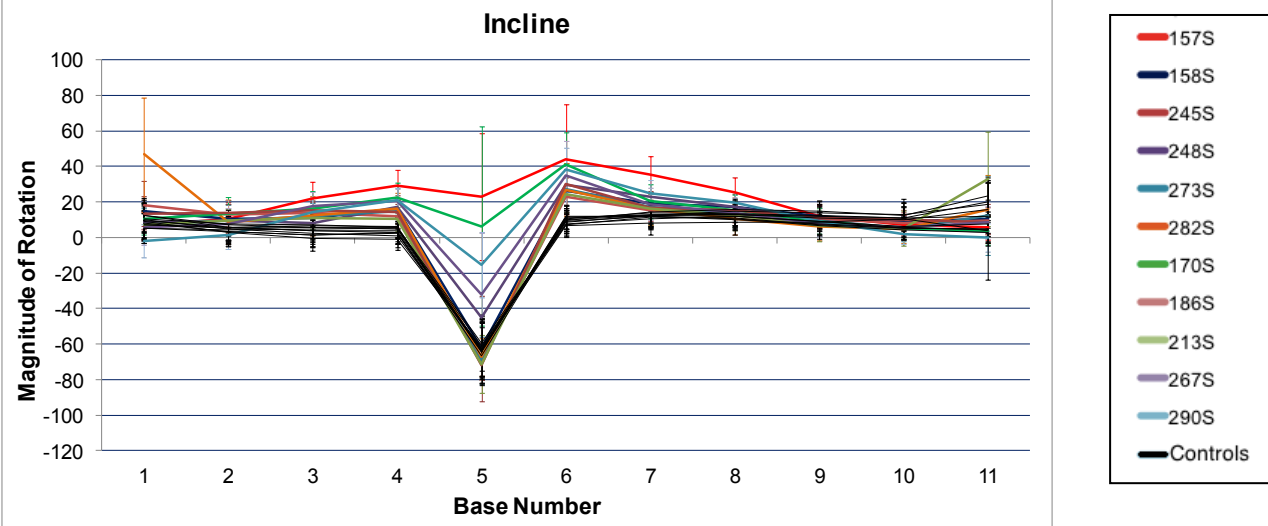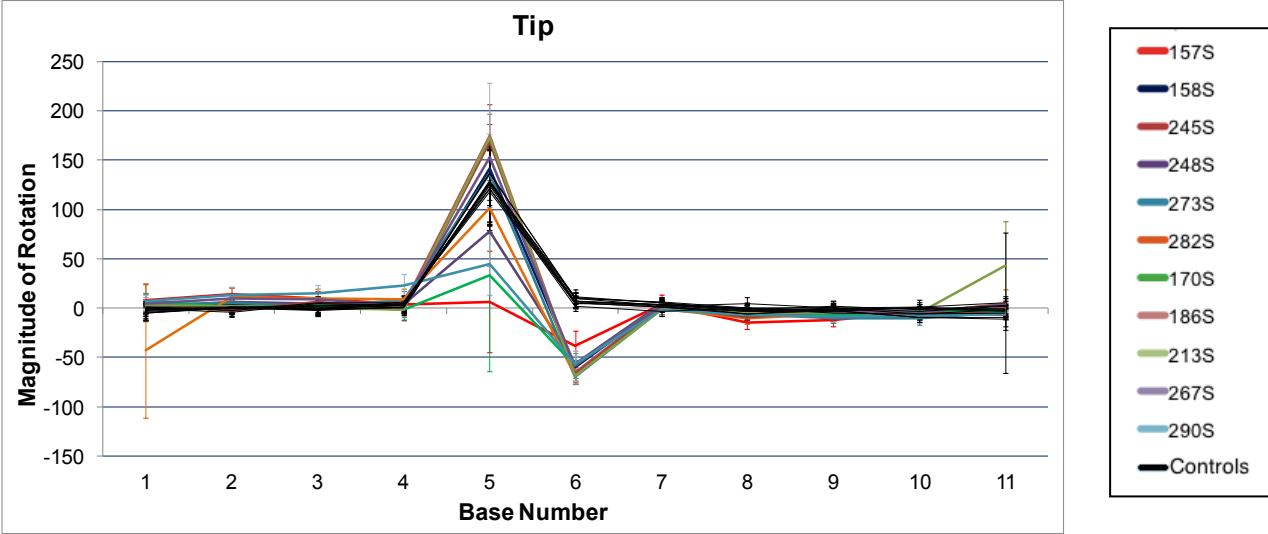

Supplementary Figure 2.

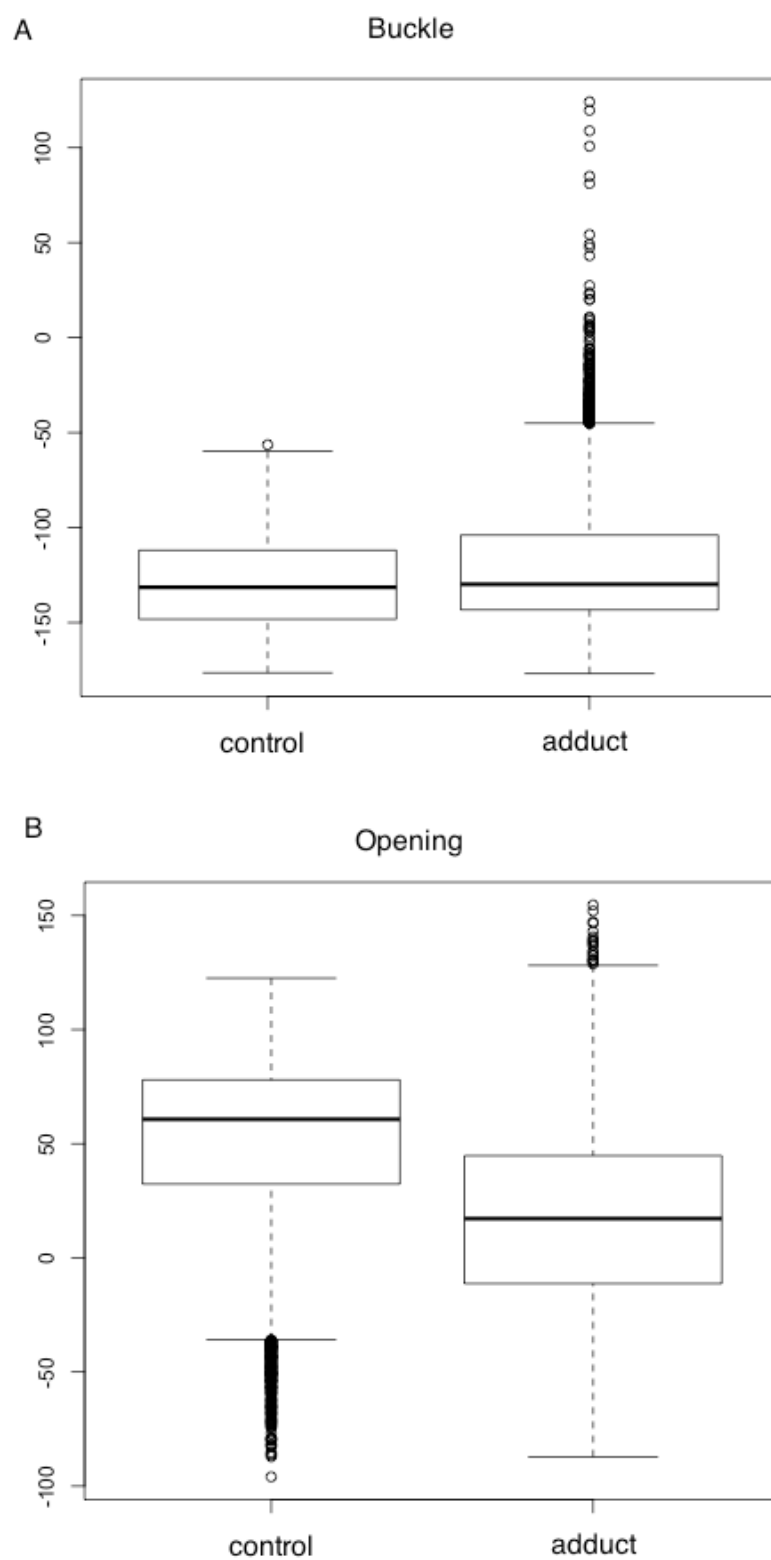

Supplement: SUPPLEMENTARY DATA [file supp_gkv910_nar-01475-f-2015-File011.pdf]
