# Supplementary material for: Base damage, local sequence context and TP53 mutation hotspots: a molecular dynamics study of benzo[a]pyrene induced DNA distortion and mutability
Source: Nucleic Acids Res. 2015 Sep 22;43(19):9133–46. doi: 10.1093/nar/gkv910 (PMC4627081; doi:10.1093/nar/gkv910)
Supplement: SUPPLEMENTARY DATA [file supp_gkv910_nar-01475-f-2015-File012.pdf]

Supplementary Table 1.

					Bond 1 Distance			Bond 2 Distance			Bond 3 Distance			Bond 1 Angle			Bond 2 Angle			Bond 3 Angle		
Codon	Adduct/ Control	I_H median	25%	75%	median	25%	75%	median	25%	75%	median	25%	75%	median	25%	75%	median	25%	75%	median	25%	75%
157	Control	4.28	2.68	6.38	2.93	2.85	3.04	2.93	2.88	2.99	2.87	2.79	2.95	164.82	157.86	170.34	166.43	160.43	171.35	164.78	158.53	170.44
	Adduct	4.40	2.79	6.40	2.94	2.86	3.05	2.96	2.91	3.03	2.86	2.80	2.95	164.23	157.24	170.17	166.16	159.95	171.19	162.11	155.09	168.14
158	Control	4.35	2.70	6.35	2.91	2.83	3.01	2.93	2.88	3.00	2.87	2.80	2.95	164.59	157.77	169.91	166.66	161.31	171.36	165.36	159.24	170.56
	Adduct	4.33	2.68	6.30	2.89	2.82	2.98	2.95	2.90	3.01	2.90	2.83	2.99	165.57	159.76	170.89	165.64	159.66	170.98	159.16	151.91	166.15
245	Control	4.38	2.73	6.34	2.91	2.83	3.01	2.93	2.88	2.98	2.86	2.79	2.94	164.53	157.93	170.08	166.68	161.01	171.41	165.02	159.10	170.34
	Adduct	4.43	2.83	6.38	2.91	2.83	3.01	2.94	2.89	3.01	2.91	2.83	3.00	161.47	152.98	168.62	165.34	159.12	170.64	156.69	147.69	164.47
248	Control	4.41	2.67	6.45	2.90	2.82	3.00	2.93	2.88	2.99	2.86	2.79	2.95	164.77	158.18	170.22	166.73	161.11	171.57	165.21	159.21	170.52
	Adduct	4.42	2.71	6.47	2.89	2.81	2.98	2.94	2.89	3.00	2.89	2.82	2.98	165.61	159.27	170.91	164.51	157.88	170.04	157.68	149.96	164.71
273	Control	4.35	2.73	6.35	2.93	2.85	3.03	2.94	2.88	2.99	2.86	2.79	2.95	164.37	157.53	169.99	165.96	159.85	170.81	164.64	158.38	170.15
	Adduct	4.43	2.75	6.39	2.90	2.82	3.00	2.95	2.90	3.01	2.88	2.80	2.96	166.09	159.88	170.99	166.95	161.65	171.74	161.06	153.97	167.43
282	Control	4.29	2.63	6.35	2.91	2.83	3.00	2.93	2.88	2.99	2.86	2.79	2.95	164.46	157.22	170.12	167.23	161.79	171.81	165.44	159.38	170.56
	Adduct	4.36	2.74	6.35	2.88	2.81	2.97	2.94	2.89	3.00	2.90	2.83	2.98	165.35	159.04	170.65	165.93	160.14	170.94	162.94	155.23	169.17
170	Control	4.33	2.75	6.33	2.90	2.82	3.00	2.93	2.88	2.98	2.87	2.80	2.95	164.32	157.12	170.01	167.03	161.61	171.65	165.32	159.47	170.58
	Adduct	4.37	2.75	6.34	2.90	2.82	2.99	2.94	2.89	3.00	2.90	2.82	2.98	164.44	157.66	170.23	166.54	160.43	171.24	161.03	153.52	167.36
186	Control	4.34	2.70	6.33	2.93	2.84	3.03	2.93	2.88	2.99	2.87	2.80	2.96	164.74	158.07	170.33	166.58	160.94	171.48	164.39	157.91	169.87
	Adduct	4.39	2.75	6.47	2.91	2.83	3.01	2.96	2.90	3.01	2.89	2.82	2.99	164.98	157.78	170.76	166.15	159.73	171.23	157.46	149.07	164.97
213	Control	4.33	2.72	6.29	2.92	2.84	3.02	2.94	2.88	2.99	2.87	2.80	2.95	165.16	158.53	170.62	166.66	161.28	171.49	164.44	157.92	170.24
	Adduct	4.38	2.79	6.41	2.87	2.80	2.96	2.95	2.90	3.01	2.91	2.83	3.01	165.20	158.46	170.67	165.26	159.12	170.49	157.01	148.74	164.59
267	Control	4.49	2.73	6.45	2.93	2.84	3.03	2.94	2.88	3.00	2.86	2.79	2.94	164.27	157.38	169.92	166.05	160.05	171.04	163.74	157.23	169.73
	Adduct	4.35	2.81	6.35	2.90	2.82	2.99	2.94	2.89	3.00	2.90	2.82	2.99	164.60	157.59	170.30	164.75	158.35	170.23	157.28	149.57	164.45
290	Control	4.40	2.76	6.38	2.92	2.84	3.02	2.93	2.88	2.99	2.87	2.80	2.95	164.96	158.15	170.35	166.42	160.87	171.35	164.11	157.51	169.68
	Adduct	4.44	2.82	6.45	2.90	2.83	2.99	2.94	2.89	3.00	2.87	2.80	2.94	151.56	145.32	157.56	166.58	161.12	171.46	161.47	154.65	167.69
